#  Creatine synthesis and transport during rat embryogenesis: Spatiotemporal expression of AGAT, GAMT and CT1

**DOI:** 10.1186/1471-213X-5-9

**Published:** 2005-05-26

**Authors:** Olivier Braissant, Hugues Henry, Anne-Marie Villard, Oliver Speer, Theo Wallimann, Claude Bachmann

**Affiliations:** 1 Clinical Chemistry Laboratory, University Hospital, CH-1011 Lausanne, Switzerland; 2 Institute of Cell Biology, Swiss Federal Institute of Technology, CH-8093 Zürich, Switzerland; 3 Institute of Molecular Biology, University of Zurich, CH-8057 Zürich, Switzerland

## Abstract

**Background:**

Creatine (Cr) is synthesized by a two-step mechanism involving arginine:glycine amidinotransferase (AGAT) and guanidinoacetate methyltransferase (GAMT), and is taken up by cells through a specific Cr transporter, CT1. Recently, genetic defects of this pathway have been described, that lead to Cr deficiency, neurological symptoms in early infancy and severe neurodevelopmental delay. To investigate the involvement of Cr synthesis and uptake pathways during embryonic development, we determined the spatiotemporal expression of AGAT, GAMT and CT1 during the rat embryogenesis, at the mRNA and protein level.

**Results:**

We show that AGAT and GAMT are expressed in hepatic primordium as soon as 12.5 days, then progressively acquire their adult pattern of expression, with high levels of AGAT in kidney and pancreas, and high levels of GAMT in liver and pancreas. AGAT and CT1 are prominent in CNS, skeletal muscles and intestine, where they appear earlier than GAMT. High levels of CT1 are found in epithelia.

**Conclusion:**

Our results suggest that de novo synthesis of Cr by AGAT and GAMT, as well as cellular Cr uptake by CT1, are essential during embryonic development. This work provides new clues on how creatine can be provided to developing tissues, and suggests that Cr deficiencies might induce irreversible damages already in utero, particularly on the nervous system.

## Background

Central nervous system (CNS) is the main organ affected in patients suffering from creatine (Cr) deficiency syndromes due either to AGAT, GAMT or CT1 deficiency [1-3]. As recently described, these patients present neurological symptoms in early infancy and show severe neurodevelopmental delay [4-6]. All three deficiencies are characterized by an absence, or a severe decrease, of Cr in CNS [7,8].

The Cr / phosphocreatine (P-Cr) / creatine kinase (CK) system is essential for the buffering and transport of high energy phosphates [9]. Cr is taken up by food, or synthesized endogenously by a two-step mechanism involving L-arginine:glycine amidinotransferase (AGAT) and S-adenosyl-L-methionine:N-guanidinoacetate methyltransferase (GAMT). Cr is taken up by cells through CT1, a specific Cr transporter belonging to the Na^+^-dependent neurotransmitter transporter family. In adult mammals, AGAT is predominantly expressed in kidney and pancreas, and GAMT is mainly localized in liver and pancreas. In addition, both enzymes are also expressed in various other tissues, albeit at lower levels. The highest expression of CT1 is found in kidney, heart and skeletal muscle (see [10] and references therein). Cr synthesis has been observed in CNS [11]. AGAT and GAMT mRNAs have been revealed in neurons, astrocytes and oligodendrocytes [12,13]. By contrast, CT1 has been found in neurons, oligodendrocytes and microcapillary endothelial cells, but is not detectable in astrocytes [13-18]. Cr plays an essential role in CNS, where it is involved in Na^+^-K^+^-ATPase activity, neurotransmitter release, maintenance of membrane potentials, Ca^++ ^homeostasis or restoration of ion gradients (for a review, see [10]). We have further shown recently that Cr might be involved in axonal growth [19]. Cr poorly crosses the blood brain barrier of rodents [20,21]; high doses of Cr given over a long period of treatment only partially replenish brain Cr of AGAT and GAMT deficient patients [7,8]. It has thus been suggested that the postnatal and adult CNS might depend, at least for a part of its needs, on its own Cr synthesis [13]. This is however in contradiction with the fact that CT1 deficient patients, who should express AGAT and GAMT correctly in their CNS, are nevertheless depleted in intracerebral Cr stores [22].

Little information is available on AGAT, GAMT and CT1 in embryonic development. AGAT (mRNA) and GAMT (protein) were found in whole extracts of the developing mouse embryo [23,24]. CT1 mRNA has been shown in the E14 rat embryo, in the entire neuraxis as well as in non-neural tissue [15]. The materno-fetal transport of Cr has been demonstrated [25,26]. As Cr deficiencies lead to severe developmental delay, our aim was to investigate at what time and in which tissues the system for Cr synthesis and transport is expressed during embryonic development. We determined therefore the tissue distribution of AGAT, GAMT and CT1 gene expression in rat embryos aged of 12.5, 15.5 and 18.5 days, at mRNA and protein level using in situ hybridization and immunohistochemistry respectively.

## Results

**Figure 1:**
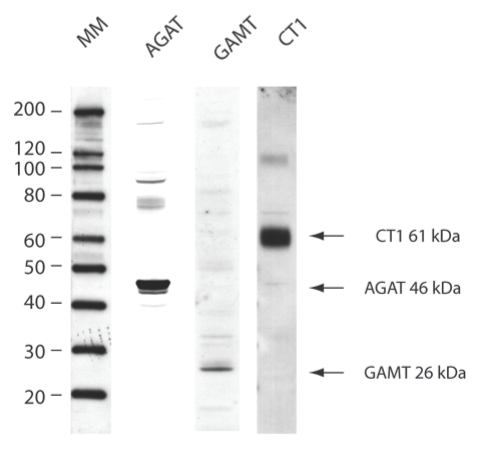
**Specificity of the anti-AGAT, anti-GAMT and anti-CT1 antibodies**. Western blot analysis of cell extract from rat kidney, by anti-AGAT, anti-GAMT and anti-CT1 antibodies. 10 μg of proteins were loaded in each lane. MM is the molecular mass marker.

**Figure 2:**
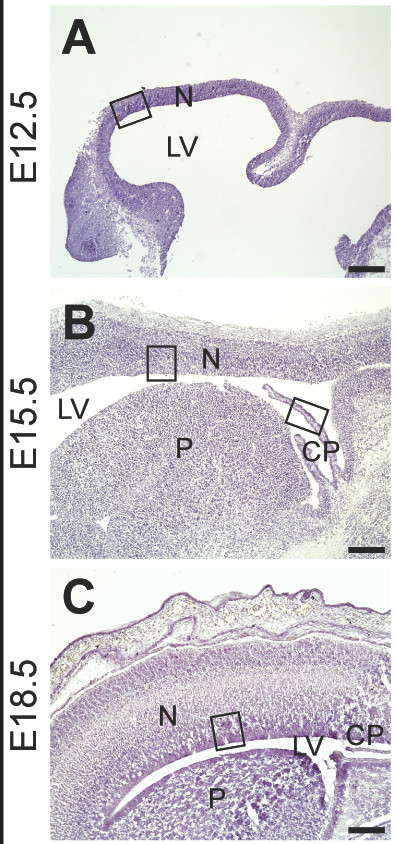
**Neocortex of the rat embryo at E12.5, E15.5 and E18.5**. Hematoxylin staining. **A**: E12.5. **B**: E15.5. **C**: E18.5. Neocortical and plexus choroid structures depicted by rectangles in **A**, **B **and **C **are enlarged in Figures 3 A,E,I; 4 B,E,H; 5 A,B,F,G,K,L; 6 A,E,I; 7 A,B,M,N; and 8 A,B. **CP**: choroid plexus; **LV**: lateral ventricle; **N**: neocortex; **P**: pallidum. Bar : 200 μm.

**Figure 3:**
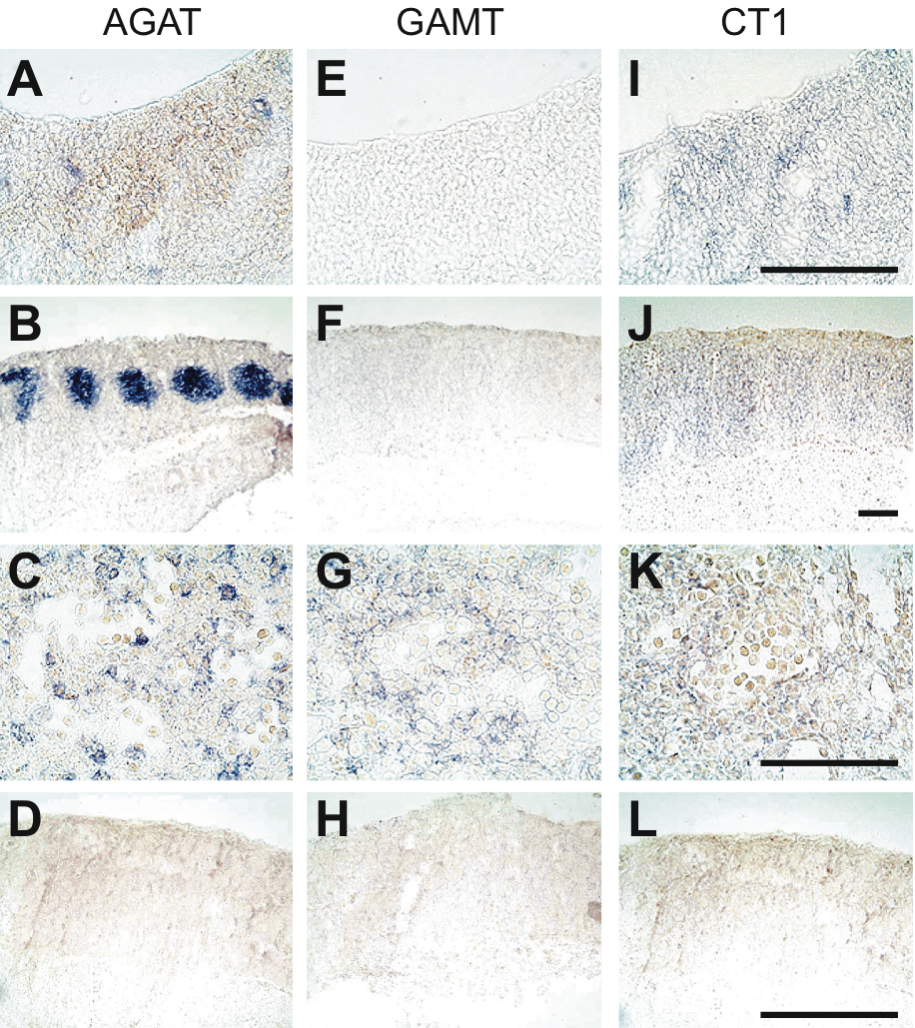
**Expression of the AGAT, GAMT and CT1 mRNAs in the E12.5 rat embryo**. In situ hybridization (mRNA, blue signal) experiments performed with antisense probes against AGAT (**A-C**), GAMT (**E-G**) and CT1 (**I-K**) mRNAs, and with the sense counterpart probes for AGAT (**D**), GAMT (**H**) and CT1 (**L**). **A,E,I **and **D,H,L**: dorsal telencephalic neuroepithelium; **B,F,J**: somites; **C,G,K**: liver. Bar : 100 μm.

**Figure 4:**
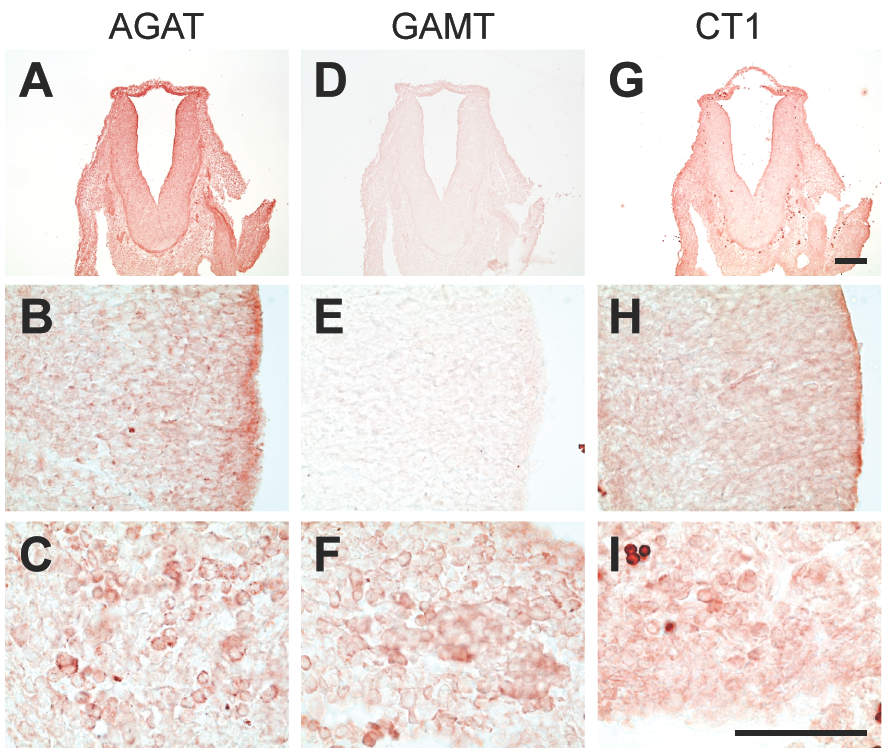
**Expression of the AGAT, GAMT and CT1 proteins in the E12.5 rat embryo**. Immunohistochemistry (protein, red signal) experiments performed with anti-AGAT (**A-C**), anti-GAMT (**D-F**) and anti-CT1 (**G-I**) antibodies. **A,D,G **: coronal section through hindbrain; **B,E,H**: dorsal telencephalic neuroepithelium; **C,F,I**: liver. Bar : **A,D,G**: 200 μm; **B,C,E,F,H,I**: 100 μm.

**Figure 5:**
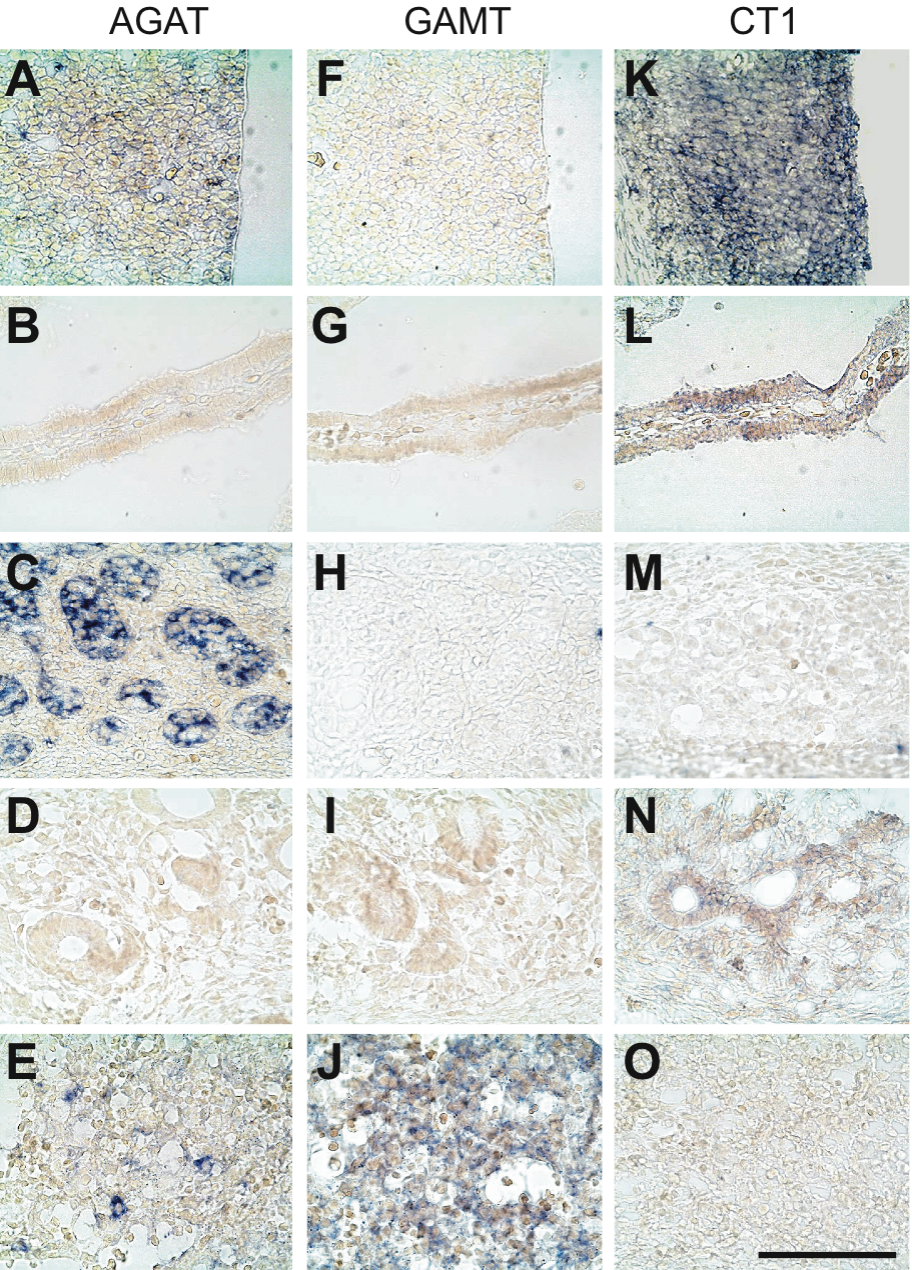
**Expression of the AGAT, GAMT and CT1 mRNAs in the E15.5 rat embryo**. In situ hybridization (mRNA, blue signal) experiments performed with antisense probes against AGAT (**A-E**), GAMT (**F-J**) and CT1 (**K-O**) mRNAs. **A,F,K**: neocortical neuroepithelium; **B,G,L**: choroid plexus; **C,H,M**: gonadal primordium; **D,I,N**: kidney; **E,J,O**: liver. Bar : 100 μm.

**Figure 6:**
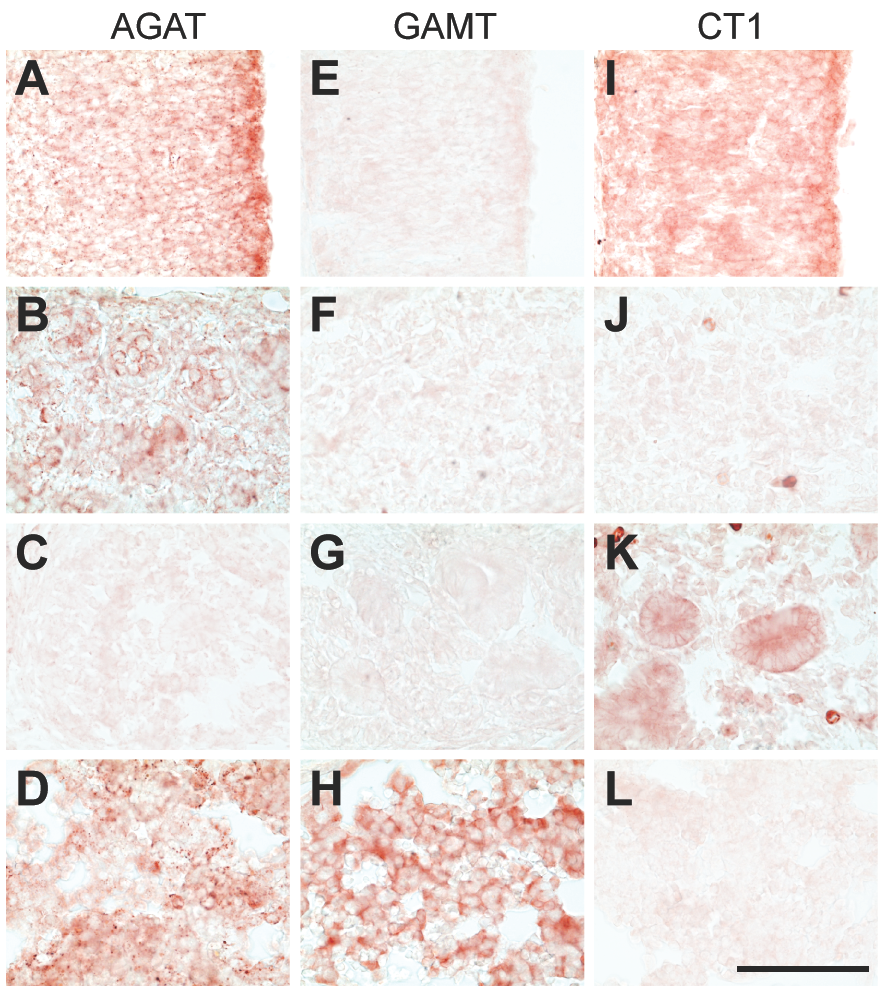
**Expression of the AGAT, GAMT and CT1 proteins in the E15.5 rat embryo**. Immunohistochemistry (protein, red signal) experiments performed with anti-AGAT (**A-D**), anti-GAMT (**E-H**) and anti-CT1 (**I-L**) antibodies. **A,E,I**: neocortical neuroepithelium; **B,F,J**: gonadal primordium; **C,G,L**: kidney; **D,H,L**: liver. Bar : 100 μm.

**Figure 7:**
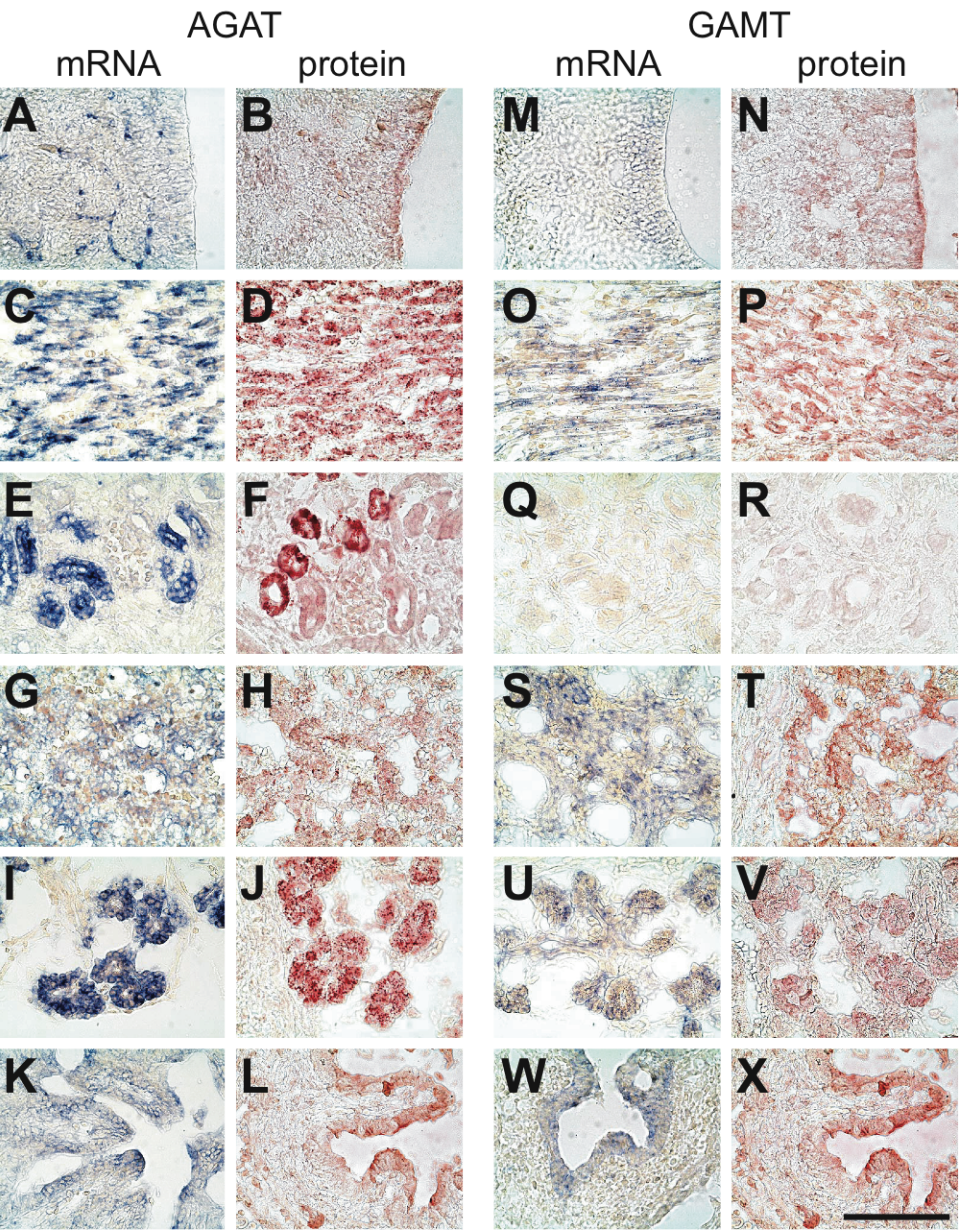
**Expression of AGAT and GAMT in the E18.5 rat embryo**. In situ hybridization (mRNA, blue signal) and immunohistochemistry (protein, red signal) experiments against AGAT (**A-L**) and GAMT (**M-X**). **A,B,M,N**: neocortical neuroepithelium; **C,D,O,P**: skeletal muscle; **E,F,Q,R**: kidney; **G,H,S,T**: liver; **I,J,U,V**: pancreas; **K,L,W,X**: intestine. Bar : 100 μm.

**Figure 8:**
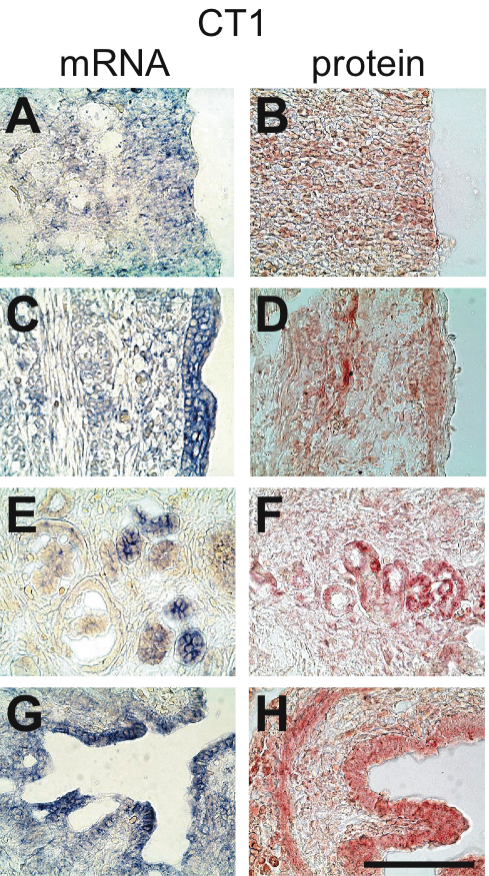
**Expression of CT1 in the E18.5 rat embryo**. In situ hybridization (mRNA, blue signal) and immunohistochemistry (protein, red signal) experiments against CT1. **A,B**: neocortical neuroepithelium; **C,D**: skin; **E,F**: kidney; **G,H**: intestine. Bar : 100 μm.

The developmental expression of AGAT, GAMT and CT1 genes was analyzed in E12.5, E15.5 and E18.5 rat embryos at the mRNA and protein levels. For each embryonic stage, patterns of AGAT, GAMT and CT1 expression were validated by **i**) the specificity of anti-AGAT, anti-GAMT and anti-CT1 antibodies (Figure 1), **ii**) the specificity of in situ hybridization probes (Figure 3 and [13]), and **iii**) the remarkable coherence between in situ hybridization (mRNA, blue) and immunohistochemistry (protein, red) stainings (see Figures 3 and 4 for E12.5, Figures 5 and 6 for E15.5, Figures 7 and 8 for E18.5). Brain structures enlarged in Figures 3 to 8 (neocortical epithelium, choroids plexus) are illustrated in Figure 2 at lower magnification.

**Figure 9:**
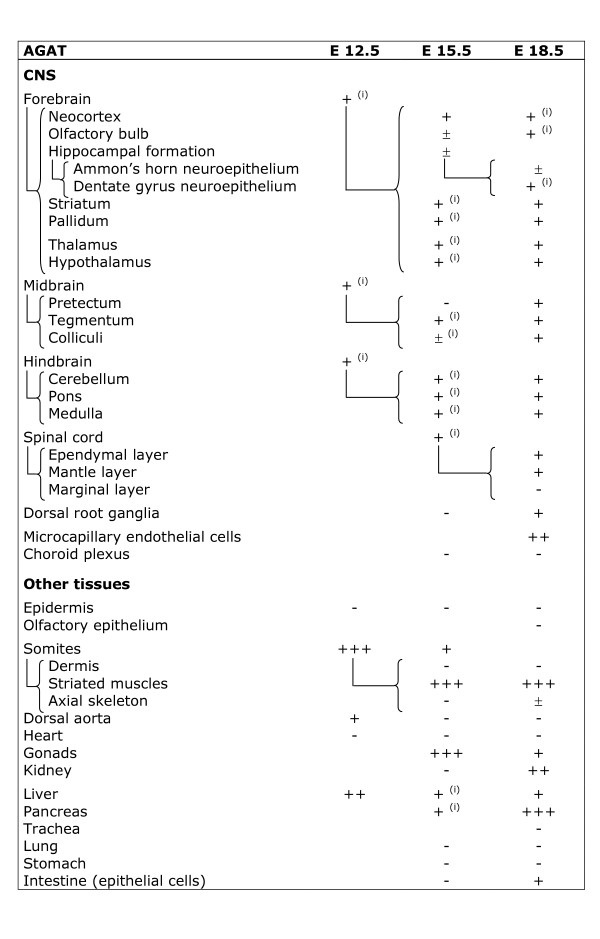
**Expression of AGAT mRNA in the main tissues of the rat embryo.** i : isolated cells ; - : absent ; ± : barely detectable ; + : moderate expression, ++ : strong expression, +++ : very strong expression. More differentiated structures of older stages are connected with the “{“ sign to the younger structure from which they originate.

**Figure 10:**
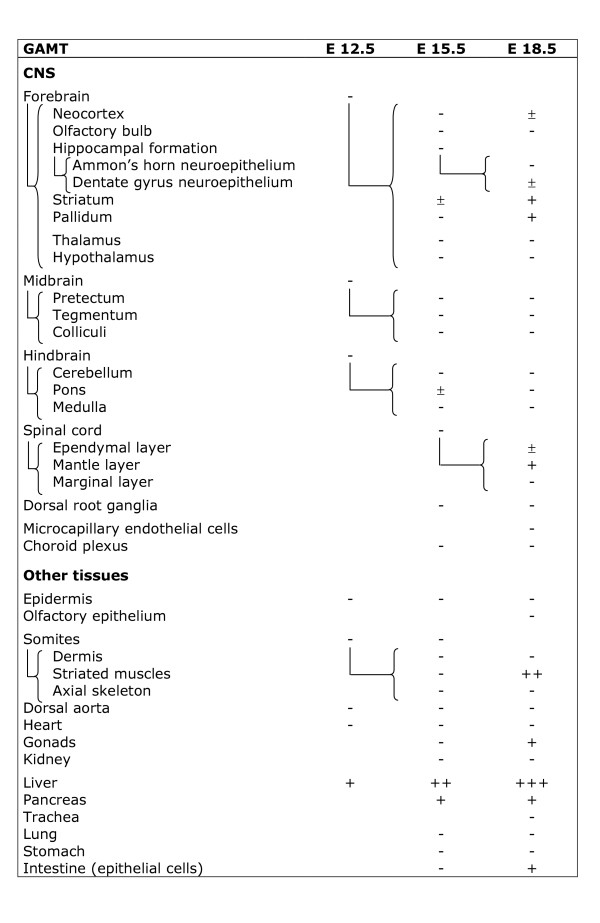
**Expression of GAMT mRNA in the main tissues of the rat embryo.** i : isolated cells ; - : absent ; ± : barely detectable ; + : moderate expression, ++ : strong expression, +++ : very strong expression. More differentiated structures of older stages are connected with the “{“ sign to the younger structure from which they originate.

**Figure 11:**
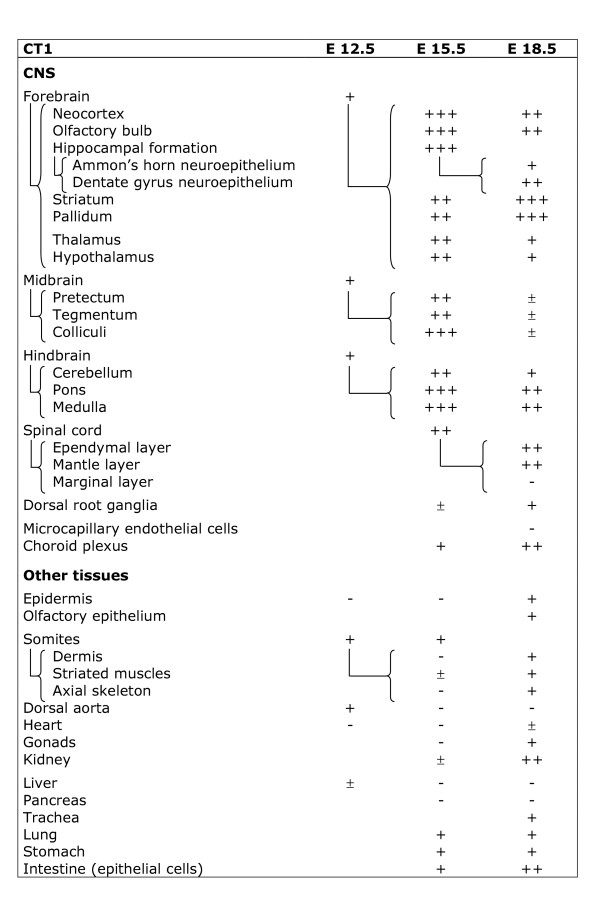
**Expression of CT1 mRNA in the main tissues of the rat embryo.** i : isolated cells ; - : absent ; ± : barely detectable ; + : moderate expression, ++ : strong expression, +++ : very strong expression. More differentiated structures of older stages are connected with the “{“ sign to the younger structure from which they originate.

### E12.5 rat embryos

AGAT was detected in all parts of the E12.5 central nervous system (Figure 9; Figures 3A; 4A and 4B). Highest levels were detected in the middle part of somites (Figure 3B) as well as in the hepatic primordium (Figures 3C; 4C). AGAT was also detected in the wall of dorsal aorta, but was not detectable in epidermis and heart (Figure 9).

GAMT at E12.5 was detectable in the hepatic primordium only (Figures 3G; 4F), with all other tissues being negative (Figure 10, Figures 3E–F; 4D and 4E).

CT1 was expressed by all parts of the E12.5 CNS (Figure 11; Figures 3I; 4G and 4E). In contrast to AGAT which was restricted to the central part of somites, CT1 was found expressed throughout the whole somites (Figure 3J). CT1 was also present in the dorsal aorta and the hepatic primordium (Figures 3K; 4I). At E12.5, CT1 was not detectable in epidermis and heart (Figure 11).

In situ hybridization control sections hybridized with the respective AGAT, GAMT and CT1 sense probes were negative (Figure 3D, 3H and 3L).

### E15.5 rat embryos

AGAT was expressed in most regions of the E15.5 CNS, with increased levels detected in isolated cells throughout the developing brain (Figure 9; Figures 5A; 6A). AGAT was not detected in choroid plexus (Figure 5B), nor in the dorsal root ganglia (Figure 9). High levels of AGAT were observed in skeletal muscles and primordia of gonads (Figures 5C; 6B), and AGAT was also detected in caudal somites (as shown earlier at E12.5, Fig. 3B), liver (Figures 5E; 6D) and pancreas, with, as in CNS, higher levels in isolated cells (Figure 9). At E15.5, AGAT could not be detected in kidney (Figures 5D; 6C), nor in all the remaining tissues observed (Figure 9).

GAMT could not be detected in most of the E15.5 CNS (Figures 5F; 6E), with the exception of striatum and pons (Figure 10). High levels of GAMT were found in liver (Figures 5J; 6H), and it was also expressed in pancreas. All the remaining tissues observed were negative for GAMT at E15.5 (Figure 10).

CT1 was strongly expressed in most of the E15.5 CNS (Figure 11; Figures 5K; 6I). CT1 was detected in choroid plexus (Figure 5L). CT1 was observed in skeletal muscles and caudal somites, as well as in kidney (Figures 5N; 6K), lung, stomach and intestine epithelial cells (Figure 11). CT1 could not be detected in gonads (Figures 5M; 6J), liver (Figures 5O; 6L) and pancreas, nor in all the remaining tissues observed (Figure 11).

### E18.5 rat embryos

AGAT mRNA was expressed in most regions of the E18.5 CNS (Figure 9). Interestingly, high levels of AGAT mRNA were detected in endothelial cells of the developing cerebral capillaries (Figure 7A), but was absent from choroid plexus (Figure 9). A high expression of AGAT, both at the mRNA and protein levels, was also detected in skeletal muscle (Figure 7C,D), kidney (Figure 7E,F) and pancreas (Figure 7I,J), while a lower level of AGAT was found in liver (Figure 7G,H) and intestine epithelial cells (Figure 7K,L). At E18.5, AGAT could not be detected in epidermis and dermis, in olfactory epithelium, trachea and lung, nor in stomach and heart (Figure 9).

GAMT was expressed in neocortex (Figure 7M,N), hippocampus, striatum, pallidum and spinal cord, but could not be detected in other structures of the E18.5 brain (Figure 10). GAMT was absent from endothelial cells of cerebral capillaries, as well as from choroid plexus. In peripheral tissues, the highest expression of GAMT, both at the mRNA and protein levels, was found in skeletal muscles (Figure 7O,P) and liver (Figure 7S,T), while it was also present in pancreas (Figure 7U,V) and intestinal epithelial cells (Figure 7W,X). GAMT could not be detected in the E18.5 kidney (Figure 7Q,R), nor in all the remaining tissues observed (Figure 10).

CT1 was highly expressed in most regions of the E18.5 CNS, both at the mRNA and protein levels (Figure 11, Figure 8A,B). CT1 was absent from endothelial cells of cerebral capillaries, but highly expressed in choroid plexus (Figure 11). Most peripheral tissues expressed CT1 (Figure 11), as illustrated for epidermis and dermis (Figure 8C,D), kidney (Figure 8E,F) and intestine epithelial cells (Figure 8G,H). However, no signal for CT1 could be detected in liver and pancreas (Figure 11).

## Discussion

### Creatine in embryonic development

The Cr / P-Cr / CK system plays an essential role in energy homeostasis during the vertebrate embryonic development, with prominent activities in tissues such as developing CNS and muscles [9]. CK genes are expressed very early in many structures of the vertebrate embryo [27,28], whereas Cr concentration between 2 and 4 mmol/kg wet weight are found in the whole rat fetus, depending on the developmental stage [29]. In CNS, Cr concentration between 5 and 8 mmol/kg wet weight have been found in rat and human fetus, depending on the gestational stage [29,30]. Parts of the developmental needs for Cr can be fulfilled by the active transport of Cr from the mother to the embryo [25,26,31]. GAMT knock-out mice, which develop biochemical alterations comparable to those found in human GAMT deficient patients, present an increase in perinatal mortality [24]. It is not known however whether alterations in Cr pathways, as found in AGAT, GAMT or CT1 deficiencies, impairs the development of the embryo.

### AGAT and GAMT for de novo synthesis of Cr in the embryo

As both substrates arginine (for AGAT) and S-adenosylmethionine (for GAMT) are available for most tissues (including liver, brain and muscle) during the mammalian embryonic development [32-34], our study suggests that the rat embryo might be able of its own Cr synthesis as soon as E12.5, in the hepatic primordium which expresses both AGAT and GAMT. From E12.5 to E18.5, both enzymes then progressively acquire their expression pattern found in aduldhood, with AGAT mainly expressed in kidney and pancreas, and GAMT preferentially localized in liver and pancreas (see [10] and references therein). As in adulthood however, many other different embryonic tissues retain low levels of AGAT and/or GAMT; we found that skeletal muscles and intestinal epithelial cells at E18.5 are equipped to synthesize their own Cr by expressing both AGAT and GAMT. A few embryonic structures express only AGAT (muscles before E18.5, regions of CNS, blood vessels) suggesting that they have to release the intermediate GAA, that has to be transported to GAMT-expressing cells for Cr synthesis to occur, as it is generally thought between kidney (AGAT) and liver (GAMT) in adult mammals. A few embryonic structures (i.e. somites at E12.5 and E15.5, skeletal muscles at E15.5 and E18.5, gonadal primordium at E15.5) showed a striking and very high expression of AGAT. This might be explained by the strong and positive regulation of AGAT, at the transcriptional level, by thyroid, growth and sex hormones [26,35,36], which do control the embryonic development of somites, skeletal muscles and gonads [37-40].

While this study shows that AGAT is expressed as soon as E12.5 in the whole CNS parenchyme and increases towards E18.5, it demonstrates also that GAMT expression in the developing CNS is delayed, and remains at low levels as compared to AGAT. Adult rat CNS appears to be able of synthesizing its own Cr by expressing AGAT and GAMT in most cell types of the brain [13]; in contrast, our work suggests that independance of CNS for its own Cr synthesis is probably limited to the end of embryogenesis and restricted to discrete regions of CNS. Thus, the embryonic brain might depend mainly on extra-CNS supply of Cr, be it of maternal origin or synthesized in other tissues of the embryo (see below).

### CT1 for the supply of Cr to embryonic tissues

From E12.5 to E18.5, most of the embryonic tissues progressively express CT1, allowing cellular Cr uptake. Notable exceptions are liver (apart from a low and transient expression at E12.5) and pancreas, which express GAMT, and endothelial cells of brain capillaries (see below). These results are in accordance with earlier work on the E14 rat embryo [15]. Highest levels of CT1 are found as soon as E12.5, in tissues requiring high amounts of Cr, like somites, skeletal muscles and CNS. In gonads, CT1 and GAMT appeared at E18.5 only, while AGAT was highly expressed at E15.5 and decreased at E18.5. This is in accordance with their respective expression in the adult reproductive tract [12,41]. CT2, a second potential Cr transporter showing 95% nucleotide identity with the CT1 coding sequence, has been found expressed in testis solely, at the RNA level [41]. However, CT2 most probably represents a pseudogene, as it cannot be fully translated into a functional protein [42], suggesting that the needs in Cr of the reproductive tract are fulfilled by CT1, as well as AGAT and GAMT.

The delayed and low level of GAMT expression during CNS embryogenesis suggests that the embryonic brain might depend on extra-CNS supply of Cr. This is supported by our results on CT1, which is expressed at high levels in the whole embryonic CNS, with a peak at E15.5. Interestingly, CT1 is expressed in the choroid plexus of the rat embryo (E15.5 and E18.5), but not of the adult rat brain [13]. In contrast, CT1 is absent from endothelial cells of brain capillaries in the embryo, while these cells express it in the adult rat CNS [13,20]. As choroid plexus differentiates earlier than brain capillaries and participates to early trophic supply for CNS [43,44], one might speculate that before angiogenesis occurs in CNS parenchyme, extra-CNS Cr is supplied from blood to cerebrospinal fluid through the choroid plexus. Cr would then be available for the whole embryonic brain through cerebrospinal fluid circulation [45] and the observed high levels of CT1 in the developing neuroepithelium, particularly in ependymal epithelium along ventricles (see below CT1 in surface epithelia exposed to amniotic fluid). As brain develops and enlarges, and CNS angiogenesis progresses, the ratio of exchange surfaces in choroid plexus and CNS microcapillaries shifts to predominance of brain microcapillaries [43-45]. Thus, at the end of embryonic development and then postnatally, Cr supply to the brain may occur preferentially at microcapillaries, with CT1 being up-regulated in capillary endothelial cells and down-regulated in choroid plexus, as in adulthood [13,20]. It should be emphasized however that supply of Cr from blood to postnatal or adult brain is very likely of less quantitative importance than intra-cerebral Cr synthesis, as astrocytes around blood brain barrier do not express CT1 [13].

At E15.5 and E18.5, CT1 was found highly expressed in all epithelia of the rat embryo being in contact with amniotic fluid, i.e. epidermis, olfactory epithelium, trachea, lung, stomach, and intestine. Cr uptake has been confirmed recently in E16 rat embryo intestine [46]. Amniotic fluid contains significant amounts of Cr (50 to 100 μM in human depending on gestational age [47]; 320 μM in rat [46]). It is produced by amniotic cells (at the surface of chorion) and the foetus, and is continuously renewed by oral intake of the foetus and excretion (urine). Amniotic fluid may thus represent an easy way to supply Cr to many structures of the embryo, through epithelial expression of CT1 in embryonic parts where vasculature is not yet fully developed.

It is known that Cr is also supplied by active transport from the mother to the embryo, accumulating in the chorioallantoic placenta and yolk sac at concentrations higher than found in maternal or fetal blood, then diffusing down its concentration gradient into the fetal circulation [25,26,31]. Thus previous studies, as well as our data, suggest that materno-fetal transport of Cr and de novo synthesis of Cr in the embryo are both necessary for a normal development to occur.

### In utero consequences of AGAT, GAMT or CT1 deficiencies

Patients suffering of AGAT, GAMT or CT1 deficiency present neurological symptoms in early infancy and show severe neurodevelopmental delay [7,22]. AGAT and GAMT deficiencies can be treated with oral Cr, which slowly replenishes brain levels of Cr [1,3-5,48-50]. Treatment of CT1 deficient patients with oral Cr does not replenish their CNS Cr level [51]. Despite developmental improvement and recovery of AGAT and GAMT deficient patients treated with Cr, sequelae to brain development and mental retardation remain [7,8]. For GAMT deficiency, this may be due to the toxicity of the GAA accumulating in CNS. Most patients with AGAT, GAMT or CT1 deficiencies are diagnosed during infancy, and significant damage to their brain occur postnatally. However, AGAT, GAMT and CT1 expression patterns during the rat embryogenesis suggests that some of the irreversible damages observed in Cr deficient patients, lacking either AGAT, GAMT or CT1, may already occur in utero.

## Conclusion

We have shown that AGAT, GAMT and CT1 are expressed by various tissues throughout the development of the rat embryo. This study suggests that de novo synthesis of Cr and Cr uptake are important for embryonic development. This work provides new clues on how creatine can be provided to developing tissues, and suggests moreover that irreversible damage observed in Cr deficient patients, lacking either AGAT, GAMT or CT1, may already occur in utero.

## Methods

### Preparation of E12.5, E15.5 and E18.5 rat embryos

All animal procedures were in compliance with the directives of the Swiss Academy of Medical Science. Sprague-Dawley pregnant female rats (Charles River Laboratories, France) were fed a standard chow formula. Embryonic stages of rat embryos were determined from the appearance of vaginal plug in pregnant females. 2 pregnant females were used for each embryonic stage (E12.5: 12.5 days of gestation, 7 embryos; E15.5: 15.5 days of gestation, 5 embryos; E18.5: 18.5 days of gestation, 5 embryos). For each embryonic stage, females were sacrificed by decapitation, and embryos were removed from uterus, rinsed in diethylpyrocarbonate (DEPC)-treated PBS and fixed for 15 h at room temperature in 4% paraformaldehyde in DEPC-treated PBS. Subsequently, embryos were cryoprotected at 4°C in 12% and 18% sucrose in DEPC-treated PBS for 18 h and 24 h respectively, then embedded in tissue-freezing medium (Jung, Nussloch, Germany) and frozen in liquid nitrogen cooled isopentane. Embryos were stored at -80°C until used for cryosections.

### In situ hybridization and immunohistochemistry

Partial cDNAs of the rat sequences AGAT (nt 182-1314, Gene Bank accession number U07971), GAMT (nt 131-734, Gene Bank J03588) and CT1 (nt 901-2544, Gene Bank NM_01738) were used to synthesize antisense and sense digoxigenin-labeled AGAT, GAMT and CT1 riboprobes as described previously [13]. 12 μm thick cryosections (Leica CM 1800) were prepared for each embryonic stage, which were analyzed by a sensitive technique of non-radioactive in situ hybridization [52]. Briefly, cryosections were postfixed 10 min in 4% paraformaldehyde in DEPC-treated PBS, washed 2 × 15 min in PBS containing 0.1% fresh DEPC and equilibrated 15 min in 5 × SSC. Sections were hybridized (58°C for 40 h in 5 × SSC, 50% formamide and 40 μg/ml salmon sperm DNA) with the digoxigenin-labeled antisense and sense riboprobes (400 ng/ml) for rat AGAT, GAMT and CT1. Sections were then washed (30 min in 2 × SSC at room temperature, 1 h in 2 × SSC at 65°C, 1 h in 0.1 × SSC at 65°C) and stained with alkaline phosphatase-coupled anti-digoxygenin antibody (Roche, Basel, Switzerland) using nitroblue tetrazolium and 5-bromo-4-chloro-3-indolyl-phosphate. The specificity of hybridization was ascertained by the use of sense probes for AGAT, GAMT and CT1 genes having the same length, GC content and digoxigenin incorporation as their antisense counterparts. In each in situ hybridization experiment, a section hybridized with an antisense probe was always followed by an adjacent section hybridized with the corresponding sense control probe. After staining, sections were dehydrated and mounted (Eukit, O. Kindler Co, Freiburg, Germany).

AGAT, GAMT and CT1 proteins were detected with rabbit polyclonal antibodies, that were made through injection of the following antigenic peptides: AGAT N-terminal amino acids (aa) 62-77 and C-terminal aa 410-423(SwissProt, accession number P50442); GAMT aa 27-227(SwissProt, accession number P10868); and CT1 N-terminal aa 15-29 (SwissProt, accession number P28570). Specific immunoglobulins against AGAT, GAMT and CT1 were obtained by peptide affinity chromatography. These antibodies recognize specific bands for AGAT (46 kDa), GAMT (26 kDa) and CT1 (61 kDa) respectively, by western blotting experiments (Figure 1). AGAT, GAMT and CT1 proteins were analyzed by immunohistochemistry on 8 μm thick cryosections using the 3 polyclonal antibodies described above. Briefly, cryosections were postfixed for 1 h in 4%PFA in PBS. Endogenous peroxidase activities were bleached using 1.5% H_2_O_2 _in PBS for 15 min. After blocking 1 h in 1% bovine serum albumin in PBS, anti-AGAT, anti-GAMT and anti-CT1 antibodies were incubated for 1 h at room temperature in the same buffer. Their detection by peroxidase staining was performed using the Histostain Plus Kit (Zymed Laboratories Inc) with aminoethyl carbazole and H_2_O_2_.

The expression patterns of AGAT, GAMT and CT1 genes observed during the embryonic development were considered specific and validated by (**i**) the excellent correlation, in the multiple embryonic tissues analysed, between the signals observed at the mRNA level by in situ hybridization and at the protein level by immunohistochemistry, (**ii**) the negative signals observed with the in situ hybridization sense probes (see Figure 3 and [13]), and (**iii**) by the absence of any labelling in immunohistochemical controls without primary antibodies or in presence of pre-immune serum (data not shown).

Sections were observed and photographed on an Olympus BX50 microscope equipped with a DP-10 digital camera (Olympus Opticals, Japan). Structures were identified according to [53-55], after staining of sections by hematoxylin (see Figure 2). In Figures 9,10 and 11 semi-quantitative levels of AGAT, GAMT and CT1 mRNA were determined based on ISH experiments, as described [56]. Levels of transcripts observed by optical microscopy are indicated by - and + signs which however do not represent a strict linear mesure of mRNA.

## List of abbreviations used

AGAT: arginine:glycine amidinotransferase; CK: creatine kinase; CNS: central nervous system; Cr: creatine; CT1: creatine transporter 1; DEPC: diethylpyrocarbonate; GAMT: guanidinoacetate methyltransferase; P-Cr: phosphocreatine

## Authors' contributions

OB conceived of the study, wrote the manuscript and performed immunohistochemistry experiments. HH designed and characterized the anti-GAMT and anti-CT1 antibodies, performed the western blotting experiments and participated in the writing of the manuscript. AMV carried out the in situ hybridization and immunohistochemistry experiments. OS designed and characterized the anti-AGAT antibody, and participated in the writing of the manuscript. TW and CB participated in the writing of the manuscript. All authors read and approved the final manuscript.
